# Temporal cortical gray matter volume relates to symptom improvement during cognitive behavioral therapy for depression

**DOI:** 10.1038/s41398-026-04545-x

**Published:** 2026-11-03

**Authors:** Konrad Schöniger, Robin Kamrla, Philine König, Esther Zwiky, Antonia Küttner, Gina Sophie Scheiwe, Julia Andreev, Johanna Elea Hamann, Annika Brückner, Sina Pletsch, Emilie Wittig, Janine Selle, Alexander Gussew, Andreas Deistung, Oliver Tüscher, Walter Alexander Wohlgemuth, Ronny Redlich

**Affiliations:** 1 Department of Psychology, Martin Luther University Halle-Wittenberg, Halle, Germany; 2 German Center for Mental Health (DZPG), Halle-Jena-Magdeburg, Halle, Germany; 3 Center for Intervention and Research on adaptive and maladaptive brain circuits underlying mental health (C-I-R-C), Halle-Jena-Magdeburg, Halle, Germany; 4 University Clinic and Outpatient Clinic for Radiology, University Hospital Halle, University Medicine Halle, Halle, Germany; 5 Halle MR Imaging Core Facility, Medical Faculty, Martin Luther University Halle-Wittenberg, Halle, Germany; 6 Department of Psychiatry, Medical Faculty, Martin Luther University Halle-Wittenberg, Halle, Germany; 7 Institute for Translational Psychiatry, University of Münster, Münster, Germany

**Keywords:** Neuroscience, Predictive markers, Prognostic markers

## Abstract

Cognitive behavioral therapy (CBT) is a first-line treatment for depression, yet treatment responses vary considerably. Understanding neurobiological factors associated with symptom improvement may help elucidate mechanisms underlying therapeutic change. This study investigated whether baseline cortical gray matter volume (GMV) is associated with reductions in depressive symptom severity during CBT. Therefore, *n* = 52 patients with unipolar affective disorders and at least mild depressive symptoms (according to BDI-II) and *n* = 42 healthy controls underwent clinical assessments and structural MRI at baseline; depressive symptom severity was reassessed after six months of outpatient CBT. Multiple regression analyses examined associations between pre-treatment cortical GMV and reductions in depressive symptoms under false discovery rate (FDR) correction. Additional models included anxiety, perceived stress, and childhood trauma as predictors. Compared to controls, patients exhibited significantly higher depressive symptom severity at baseline and greater symptom reduction over time (all *p* < 0.001). Within patients, baseline GMV of the right middle temporal gyrus (MTG) was significantly associated with symptom reduction (baseline–follow-up; β = 0.55, *p*_FDR_ = 0.033). Right MTG volume accounted for additional variance in symptom reduction beyond established clinical variables (Δ*R*² = 0.15). Group differences in GMV did not remain significant following FDR correction. These findings suggest that temporal gyrus GMV is associated with symptom improvement following CBT in unipolar depression, extending the focus beyond traditionally emphasized limbic regions. Preserved temporal morphology may support cognitive and emotional processes engaged during CBT, underscoring the relevance of temporal cortical structure for understanding the neurobiological correlates of psychotherapy response.

## Introduction

Depressive disorders represent one of the leading causes of mental health-related burden worldwide [1, 2], with a global prevalence of approximately 6% [3]. Although depressive disorders can manifest in highly diverse phenotypes, they share common features such as a tendency toward relapse and impairments in emotional and cognitive functioning [4]. Cognitive behavioral therapy (CBT) is an evidence-based psychotherapy for depression, is widely recommended in clinical guidelines as a first-line psychological treatment, and produces enduring reductions in depressive symptoms [5]. However, treatment response to CBT remains heterogeneous: while many patients experience substantial improvement, a considerable proportion fail to achieve full remission [6]. Identifying factors associated with treatment response prior to therapy may improve patient–treatment matching and clinical outcomes, and investigating the neurobiological mechanisms underlying such variability may help inform more personalized psychotherapy approaches.

Neuroimaging research has consistently identified structural brain alterations in depression, particularly within cortico-limbic regions such as the hippocampus, amygdala, anterior cingulate cortex, and orbitofrontal cortex, which are implicated in emotional processing and regulation [7–9]. However, structural correlates of depression identified through case-control comparisons have proven to be subtle and inconsistent across studies. In their large meta-analysis (*N* > 10,000), Ho et al. [10] reported cortical thinning in adults with major depressive disorder relative to controls, with effect sizes ranging from d = 0.10–0.14 [10]. Most recently, a multi-cohort investigation confirmed that reliable structural correlates of depression are restricted to a small set of regions, including the frontal cortex, anterior cingulate, and insula, while several classically implicated regions, including subcortical structures, showed no consistent association [11]. Together, these findings indicate that structural brain differences between depressed patients and healthy individuals are real but modest in magnitude, likely reflecting substantial between-subject heterogeneity within diagnostic categories. This heterogeneity motivates a shift in emphasis from case-control comparisons toward individual-differences approaches that examine how structural variation within patients relates to clinically meaningful outcomes, and, building on these findings, studies have increasingly examined neural factors associated with treatment response in depression. Both structural (e.g., [12]) and functional (e.g., [13]) imaging studies have reported brain correlates of psychotherapy outcome, particularly within neural networks implicated in emotion regulation, cognitive control, and self-referential processing [14–17]. Several studies have used functional MRI data to predict treatment response [18–20], while others have focused on structural measures, such as regional brain volume [14], cortical surface metrics [21], or structural connectivity [22]. However, findings across studies remain inconsistent, likely reflecting small sample sizes, methodological heterogeneity, and diagnostic variability.

Research on the neural structural correlates of psychotherapy response has largely focused on subcortical and limbic regions, such as the amygdala, hippocampus, and anterior cingulate cortex, as well as prefrontal regions implicated in cognitive control and emotion regulation (e.g., [12, 23]). Comparatively less attention has been directed toward cortical structures that may also play an important role in psychotherapy-related change and may contribute to variability in treatment response. In particular, regions within the temporal and parietal cortex are involved in processes such as social cognition, autobiographical memory, and the integration of emotional and cognitive information [24–27]. These functions are frequently engaged during CBT and may therefore be relevant for understanding individual differences in therapeutic change.

A growing body of work indicates that depressive symptom improvement depends in part on a patient’s broader clinical context, including current perceived stress, stable trait anxiety, and exposure to early-life adversity [28–30]. Building on this literature, we assessed these variables and evaluated them as established predictors in the present study.

The present study aimed to identify cortical neurostructural baseline markers associated with CBT treatment outcome, expanding prior work that has largely emphasized subcortical circuitry. To this end, cortical gray matter volumes were examined as baseline correlates of subsequent reductions in depressive symptom severity over the course of six months of therapy in patients with unipolar depression (MDD). By examining structural brain correlates of symptom improvement, this study seeks to contribute to the growing field of precision psychiatry and to inform neurobiologically grounded approaches to individualized treatment strategies. We hypothesized that greater baseline cortical gray matter volume (GMV) would be associated with greater subsequent symptom reduction during CBT, reflecting the possibility that greater structural integrity may be associated with greater capacity to engage in cognitive and emotional processes relevant to CBT. We were particularly interested in examining cortical GMV across the brain to identify cortical regions associated with treatment response. Specifically, the study addressed (1) whether baseline cortical GMV is associated with individual differences in symptom reduction during CBT and (2) whether cortical structural measures provide incremental value beyond established clinical predictors, including perceived stress, trait anxiety, and childhood trauma exposure.

## Materials and methods

### Participants, measures, and study design

*N* = 94 participants, including *n* = 52 depressed patients (age: *M* = 28.31 years, *SD* = 8.30; gender: 36/16 [f/m]) and *n* = 42 matched healthy controls (HC; age: *M* = 27.45, *SD* = 7.59; gender: 28/14 [f/m]), were recruited at the Department of Psychology, University of Halle between October 2022 and November 2025 prior to the beginning of CBT (MDD). HC were included as a normative comparison group to characterize baseline differences associated with depression; however, they do not constitute an active control condition and therefore cannot isolate CBT-specific treatment effects. All participants underwent structural MRI, the German Structured Clinical Interview for DSM-5 disorders (SCID-5-CV) [31], and completed clinical questionnaires, including the German Beck Depression Inventory-II (BDI-II) [32], a 21-item retrospective self-report questionnaire that assesses presence of current depression symptoms, alongside the German Perceived Stress Scale (PSS-10) [33], the trait-version of the German State Trait Anxiety Inventory (STAI-T) [34], and the German Childhood Trauma Questionnaire (CTQ) [35, 36]. All baseline MRI and clinical assessments were completed before initiating CBT.

Patients were required to meet full diagnostic criteria for a current depressive disorder according to the SCID-5-CV [31], which served as the primary diagnostic assessment. HC were only included if they did not meet criteria for any disorder according to the SCID-5-CV. In addition, a BDI-II score >9, indicating at least mild depression according to the Center for Cognitive Therapy guidelines for patients with affective disorders [37], was required for inclusion in the patient group and served as an exclusion criterion for HC at baseline. A medication load index for each patient was computed following the approach of Hassel [38]. Exclusion criteria for all participants were any history of neurological (e.g., concussion, stroke, tumor, neuroinflammatory disease) or medical conditions (e.g., cancer, chronic inflammatory or autoimmune disorders, cardiovascular disease), as well as current or past substance-use disorders.

All patients received standardized outpatient CBT delivered by licensed psychotherapists or therapists in training in the outpatient units of either the University of Halle-Wittenberg or the Halle Institute for Psychological Psychotherapy training. HC were recruited through public notices and newspaper announcements. Six months after initial recruitment, participants were invited to complete the follow-up assessment consisting of the BDI-II. The mean interval between baseline and follow-up assessment was *M* = 27.70 weeks (*SD* = 2.08). Information on the study sample, including psychiatric comorbidities, is presented in Table 1. All participants received financial compensation. The study was conducted in compliance with the Declaration of Helsinki, was approved by the local Institutional Review Board, and written informed consent was obtained from all participants prior to participation.

**Table 1:** Sociodemographic and clinical sample characteristics.

	MDD (*n* = 52)	HC (*n* = 42)		
	*M* (*SD*)	*M* (*SD*)	Group comparison	*p*
**Sociodemographic characteristics**				
Gender (f/m)	36/16	28/14	χ^2^(1) = 0.07	0.791
Age	28.31 (8.30)	27.45 (7.59)	*t*(90.53) = 0.52	0.604
Years of education	13.04 (2.06)	13.38 (2.17)	*t*(85.70) = 0.78	0.439
**Longitudinal interval**				
Time interval in weeks	27.73 (2.08)	27.67 (2.11)	*t*(87.37) = 0.13	0.899
Number of CBT sessions	18.94 (10.90)	-	-	-
**Clinical characteristics**				
*Clinical questionnaire data*				
Depressive symptom severity at baseline	22.94 (8.73)	2.86 (2.66)	*F*(1,90) = 205.71	<0.001
Depressive symptom severity at follow-up	14.75 (11.21)	2.07 (2.28)	*F*(1,90) = 53.08	<0.001
Anxiety	55.54 (7.29)	33.50 (7.36)	*F*(1,90) = 209.07	<0.001
Perceived stress	35.77 (4.46)	21.83 (4.99)	*F*(1,90) = 208.35	<0.001
Childhood trauma experiences	40.67 (10.80)	33.67 (10.25)	*F*(1,90) = 10.22	0.002
*Medication load index*				
0 = absent	42 (80.77%)	-	-	-
1 = low	9 (17.31%)	-	-	-
2 = high	1 (1.92%)	-	-	-
*DSM-V diagnosis*				
MDD, recurrent	34 (65.38%)	-	-	-
MDD, single episode	8 (15.38%)	-	-	-
Dysthymia	9 (17.31%)	-	-	-
Other depressive disorder	1 (1.92%)	-	-	-
*Degree of remission*				
In partial remission	2 (3.85%)	-	-	-
Acute	50 (96.15%)	-	-	-
Comorbidity (yes/no)	31/21	-	-	-

Groups included depressed patients (MDD) and healthy controls (HC); clinical diagnoses were assessed using the German Structured Clinical Interview for DSM-5 disorders [31]. Group differences in age, years of education, and assessment interval were assessed using Welch’s *t*-tests. All other continuous variables were compared between groups using analysis of covariance models adjusted for age and gender. Gender was compared using a χ2-test. Depressive symptom severity was measured with the German Beck Depression Inventory-II [32] at baseline and at follow-up after cognitive behavioral therapy (CBT). At baseline, trait anxiety was assessed using the German State-Trait Anxiety Inventory [34], perceived stress using the German Perceived Stress Scale [33], and childhood trauma experiences using the German Childhood Trauma Questionnaire [35]. Medication load index for each patient was computed following the approach of Hassel [38].

### MRI procedures

All MR data were collected using a 3 T scanner (Magnetom Vida 3 T, Siemens, Erlangen, Germany) with a 64-channel head/neck coil. Whole-head T1-weighted anatomical images were collected with a magnetization-prepared rapid gradient-echo (MP-RAGE) sequence, isotropic voxel size = 0.8 mm, repetition time = 2400 ms, echo time = 2.24 ms, inversion time=1030 ms, flip angle = 8°, acquisition matrix = 320 × 300 × 208, and GRAPPA with R = 2 and 32 reference lines, yielding an acquisition time of 6:38 min.

Structural MRI data were processed using the automated cortical reconstruction and volumetric segmentation pipeline implemented in FreeSurfer (version 5.3). Preprocessing was performed using the standard *recon-all* stream with default parameters, which includes intensity normalization, skull stripping, and cortical surface reconstruction. Cortical regions were parcellated according to the Desikan-Killiany atlas [39]. GMV of all 68 cortical regions (34 per hemisphere) was extracted and used for subsequent analyses. Segmentation quality was assessed through visual control, statistical outlier screening, and quantitative assessment of cortical surface reconstruction quality using the FreeSurfer Euler number, an established index of reconstruction quality from cortical surface topology [40], with lower values indicating a greater number of topological defects.

### Statistical analysis

All statistical analyses were conducted using R (v4.5.0) and RStudio (v2023.06.1). Standard statistical analyses were performed using the *stats* (v4.5.0) package. Elastic net models were estimated using the *glmnet* (v4.1.10) [41] and *caret* (v7.0.1) [42] packages. Statistical significance was set at α = 0.05; all analyses were performed two-sided for exploratory reasons. For analyses of GMV data, resulting *p*-values were corrected for multiple comparisons using the Benjamini–Hochberg false discovery rate (FDR) procedure at α < 0.05 due to multiple testing. Analyses were conducted in two sequential steps, the first including all participants, the second including only MDD patients.

First, to characterize baseline differences between MDD and HC, group comparisons of sociodemographic, clinical, cortical GMV, and MRI reconstruction quality data were performed using Welch’s *t*-tests and additional Mann-Whitney *U* tests for sociodemographic variables and Euler numbers due to the non-normal distribution as confirmed by group-wise Shapiro-Wilk tests, as well as linear models with group as the primary predictor (ANCOVA). Treatment-related change in depressive symptoms was assessed separately within the MDD group by comparing pre- and post-treatment BDI-II scores using a paired-samples *t*-test, given normality of pre–post differences (Shapiro-Wilk *p* = 0.898). In addition, a mixed-design group × time analysis of variance (ANOVA) was computed to examine main and interaction effects on depressive symptom severity across time.

Second, in MDD patients, associations between baseline clinical characteristics and cortical GMV with reductions in depressive symptom severity over the course of 6 months of CBT were examined. Therefore, multiple linear regression analyses were conducted using total and percentage reductions in BDI-II scores as outcome variables. Age, gender, and medication load index were included as covariates of no interest in all analyses. In all analyses involving cortical GMV measures, total intracranial volume (TIV) was additionally included as a covariate of no interest to account for individual differences in head size. All continuous variables were z-standardized prior to analysis.

Regional GMV for each of the 68 cortical regions was entered separately as the predictor of interest, yielding 68 individual regression models (one per cortical region). Model assumptions (residual normality, homoscedasticity, multicollinearity, and case influence) were assessed for all regression models; full details are reported in the Supplemental Material. Sensitivity analyses, including the total FreeSurfer Euler number as an additional covariate in the primary regression models to assess MRI reconstruction quality, are reported in the Supplemental Material.

In an exploratory analysis, BDI-II symptoms were further divided into cognitive and affective-somatic domains based on the proposed two-factor structure by Beck et al. [43]. A linear mixed-effects model was used to test whether the association between identified significant regions and symptom improvement differed between domains. Results are provided in the Supplemental Material. To examine whether treatment response status (defined as a ≥ 50% reduction in BDI-II scores) moderated the association between cortical GMV and treatment response, an additional moderation analysis was conducted for identified significant predictors. Thus, a separate multiple linear regression model was estimated including interaction terms between regional GMV and response status. Additionally, patients who improved versus those whose symptoms worsened during treatment were compared (see Supplemental Material).

To evaluate whether cortical GMV explained additional variance in symptom reduction beyond established clinical predictors, hierarchical multiple regression analyses were performed. In the first model step, baseline perceived stress, trait anxiety, and childhood trauma exposure were entered as predictors. In the second model step, regional cortical GMV was added. Incremental variance explained by cortical volume was quantified using the change in explained variance (Δ*R*²).

Finally, in an exploratory post hoc analysis, elastic-net regularized linear regression was used to examine out-of-sample predictive performance of both baseline clinical and neuroanatomical measures with respect to depressive symptom reduction. Total BDI-II reduction served as the outcome variable. Baseline childhood trauma, perceived stress, trait anxiety, gender, age, estimated total intracranial volume (TIV), and medication load were included as unpenalized covariates, while cortical GMV measures were entered as penalized predictors. Model training and evaluation were conducted using repeated nested five-fold cross-validation to reduce potential estimation bias, with hyperparameter tuning restricted to inner training folds. Predictive performance was quantified using cross-validated coefficients of determination (*R*²) and root mean squared error (RMSE).

## Results

### Group differences at baseline

Data from *n* = 52 MDD patients and *n* = 42 HC were analyzed in this study (see Table 1). While the groups were comparable in terms of age, gender distribution, or years of education (Welch’s *t*-tests: *p* ≥ 0.439; Mann-Whitney *U* tests: *p* ≥ 0.433), ANCOVAs revealed significantly higher levels of trait anxiety and childhood trauma experiences in MDD compared with HC (*p* ≤ 0.002). Regarding FreeSurfer-derived baseline cortical volume measures, nominal group differences were observed in the left caudal middle frontal gyrus, right entorhinal cortex, right rostral anterior cingulate gyrus, and right inferior parietal gyrus (*p* ≤ 0.039, see Supplemental Table 1). However, none of these differences remained significant after FDR-correction (*p*_FDR_ ≥ 0.239). Regarding MRI reconstruction quality, no group differences were observed (*p* ≥ 0.178, see Supplemental Results and Supplemental Table 2).

### Depressive symptom severity across timepoints in patients and controls

**Fig. 1:**
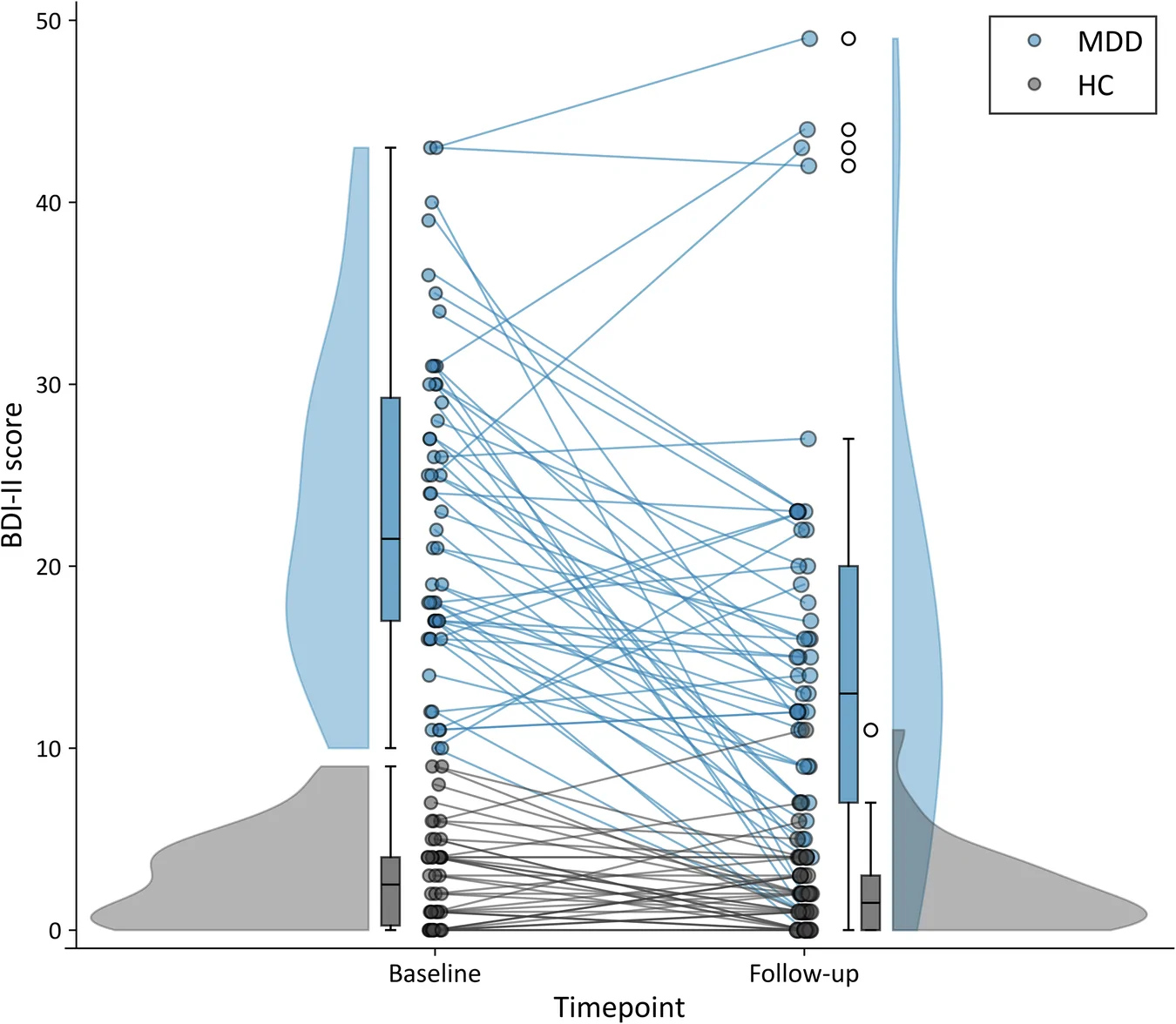
Depression symptom changes in patients and healthy controls. Longitudinal changes in depressive symptom severity measured with the German Beck Depression Inventory-II [32] of *n* = 52 depressed patients (MDD) and *n* = 42 healthy controls (HC). Each dot represents an individual participant; lines indicate changes from baseline to follow-up. Boxplots and raincloud plots show group-level distributions.

At baseline, BDI-II scores differed between MDD patients and HC, with patients showing higher symptom severity after adjustment for age and gender (*F*(1,89) = 205.71, *p* < 0.001). At follow-up, BDI-II scores remained higher in MDD patients than in HC after adjustment for age and gender (*F*(1,89) = 53.08, *p* < 0.001). Within the MDD group, BDI-II scores decreased significantly from pre- to post-treatment (mean change [*SD*] = 8.19 [11.28], *t*(51) = 5.24, *p* < 0.001), indicating a significant reduction in depressive symptom severity over the course of CBT. Individual changes in BDI-II scores from baseline to follow-up are presented in Fig. 1. The corresponding group × time ANOVA is reported in the Supplemental Results.

### Prospective associations between baseline measures and depressive symptom reduction

Within the MDD group, baseline perceived stress and childhood trauma showed positive associations with baseline depressive symptom severity (both p < 0.0024; Intercorrelations among study variables are presented in Table 2). Among the clinical variables examined, only baseline perceived stress was significantly associated with reductions in BDI-II scores over the course of treatment (β = 0.32, *p* = 0.019).

**Table 2:** *Intercorrelations between clinical measures and sociodemographic variables in n* = *52 depressed patients*.

Variable	BDI-II pre-CBT	BDI-II post-CBT	STAI-T	PSS-10	CTQ	Age	Education
BDI-II pre-CBT	—						
BDI-II post-CBT	0.38	—					
STAI-T	0.41**	0.11	—				
PSS-10	0.63**	0.16	0.37	—			
CTQ	-0.08	0.17	-0.06	-0.03	—		
Age	-0.12	0.04	0.05	0.07	0.19	—	
Education	-0.26	-0.09	-0.15	0.05	0.01	0.10	—

Pearson correlation analyses were conducted. Depressive symptom severity was measured with the German Beck Depression Inventory-II (BDI-II) [32] at baseline and again after 6 months of CBT, while additionally, only at baseline, trait anxiety was assessed using the German State-Trait Anxiety Inventory (STAI-T) [34], perceived stress using the German Perceived Stress Scale (PSS-10) [33], and childhood trauma experiences using the German Childhood Trauma Questionnaire (CTQ) [35]. Education refers to years of education.

*p*-values were evaluated using a Bonferroni-adjusted significance threshold of α = 0.0024.

*p < 0.0024, ** *p* <0.001.

**Fig. 2:**
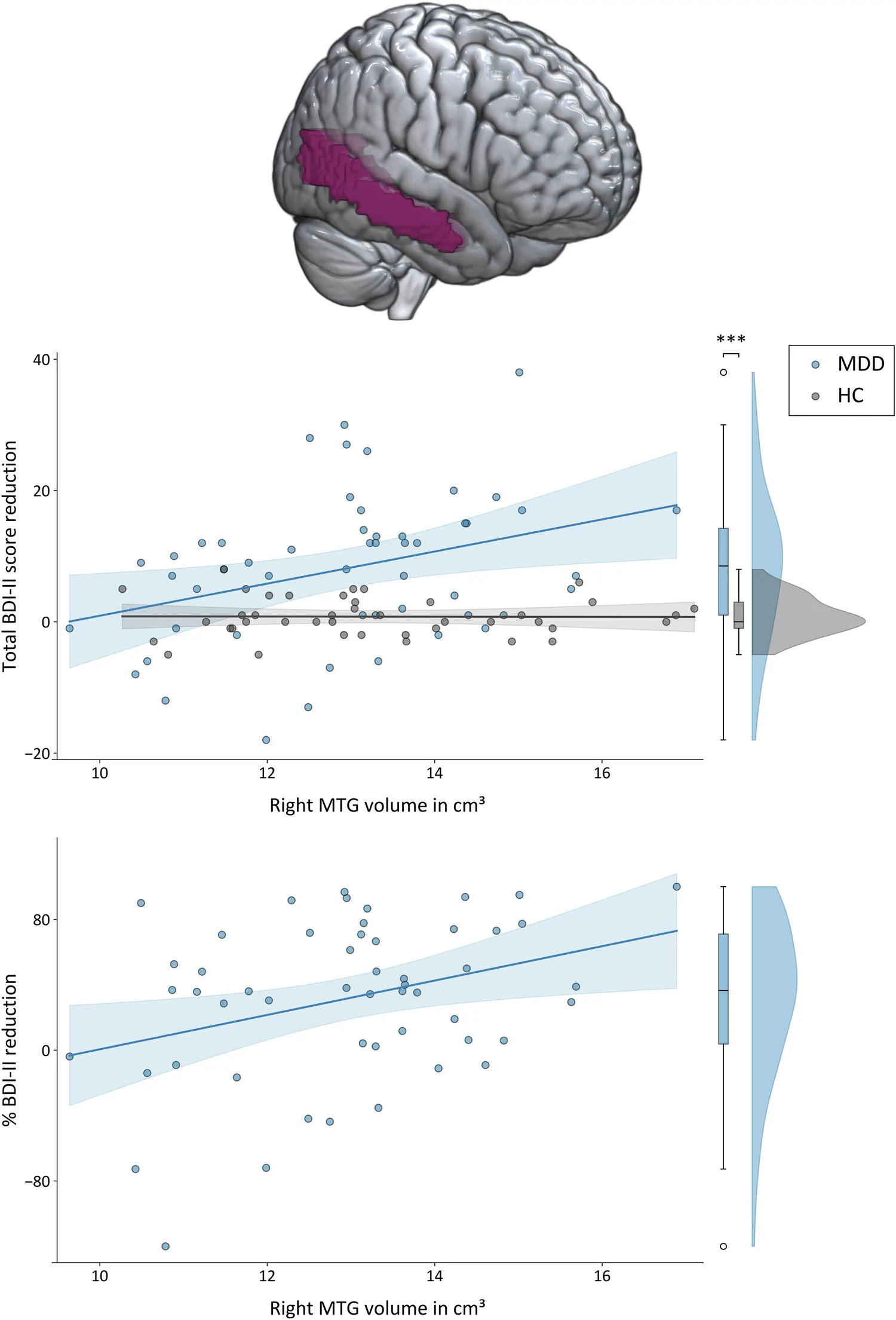
Associations between depression severity reductions and right middle temporal gyrus volume. Associations between middle temporal gyrus (MTG) gray matter volume of the right hemisphere (purple; at the top), total (middle), as well as percentage (bottom) reduction in depressive symptom severity measured with the German Beck Depression Inventory-II [32] in depressed patients (MDD) and healthy controls (HC). Lines show the fitted ordinary least squares regressions (top: β_Patients_ = 0.55, bottom: β = 0.49); shades represent the 95% CI of the regression mean. Rainclouds show the distribution of scores (wider areas indicate higher concentration), with boxplots summarizing the median and interquartile range. *** *p* < 0.001.

Among cortical measures, baseline GMV of the right middle temporal gyrus (MTG) was significantly associated with total BDI-II reduction after FDR correction (total reduction: β = 0.55, *p*_FDR_ = 0.033; percentage reduction: β = 0.49, *p*_FDR_ = 0.063; see Fig. 2). Left MTG volume showed only a trend-level association (*p*_FDR_ ≥ 0.196). No other cortical regions were significantly associated with symptom reduction (all *p*_FDR_ ≥ 0.05).

Treatment response status (≥50% reduction in BDI-II scores) did not moderate the association between MTG volume and symptom change (*p* < 0.321), although the association was descriptively stronger among responders (*n* = 19, β = 0.64) than non-responders (*n* = 33, β = 0.34).

To examine whether cortical volume explained additional variance in symptom reduction beyond established clinical predictors, hierarchical linear regression analyses were conducted, including trait anxiety, perceived stress, and childhood trauma in the first model step. When right MTG volume was added in the second step, the increase in explained variance was moderate in magnitude but did not reach statistical significance after correction for multiple comparisons (Δ*R*² = 0.15, *p*_FDR_ = 0.066).

### Post hoc analysis: out-of-sample prediction

In an exploratory post hoc analysis, out-of-sample predictive performance of both baseline clinical and neuroanatomical measures was evaluated. The combined model demonstrated modest predictive performance (*M*_*R*²_ = 0.04, 95% CI [0.00, 0.12]; *M*_RMSE_ = 12.39, 95% CI [11.01, 13.98]). Overall, baseline cortical volume measures provided limited additional predictive value beyond established clinical and demographic variables in the out-of-sample prediction setting. Given the exploratory nature of these analyses, the modest predictive performance, and the sample size, these findings should be interpreted cautiously and require replication in independent samples.

## Discussion

In this study, we investigated whether baseline GMV is linked to reductions in depressive symptom severity following six months of outpatient CBT in patients with unipolar depression. Compared to HC, MDD patients exhibited heightened levels of clinically relevant symptoms, including depression and anxiety, as well as the risk factor of childhood maltreatment. A significant depressive symptom reduction over the course of CBT was observed. The findings suggest that pre-treatment GMV of the right MTG was associated with greater symptom improvement. Importantly, the association remained substantial after accounting for established clinical variables, including baseline depressive symptom severity, anxiety, perceived stress, and childhood trauma, suggesting that right MTG morphology provides unique value for characterizing individual variability in psychotherapy response.

These findings extend prior research primarily focused on subcortical and limbic structures, such as the hippocampus and amygdala, by highlighting the potential relevance of the temporal cortex in psychotherapy outcomes. While the temporal lobe is classically associated with auditory and visual processing [9, 44, 45], the MTG is particularly implicated in the processing of complex cognitive, social, and emotional information, including semantic memory, empathy, emotion regulation, and the integration of contextual cues with emotional appraisal [24, 27, 46–48], processes often impaired in depression [49]. Functional and structural alterations in the MTG in MDD patients have been associated with deficits in these functions, alongside negative cognition and mood processing in MDD patients [9, 50, 51]. These functions are particularly relevant to CBT, which often requires patients to reinterpret social and emotional experiences, retrieve alternative cognitive frameworks, and flexibly integrate new perspectives into their thought patterns. Even though there were no group differences in MTG volume observed within this study, it may provide a neuroanatomical substrate that facilitates these processes, allowing patients to more effectively engage with cognitive restructuring and emotion regulation exercises.

Previous studies repeatedly exhibited GMV decreases in the MTG in depression (e.g., [8, 9]), which were accompanied by a progressive decrease over time, which was in turn attributed to disease progression [52]. McCurdy et al. [53] showed that GMV reduction in the right MTG is associated with involvement in CBT [53]. In this context, greater preservation of temporal cortical structure may be relevant to individual differences in treatment-related improvement. One possibility is that preserved MTG morphology supports higher-order processes involved in identifying, contextualizing, and modifying maladaptive cognitions, including negative beliefs and automatic thoughts. The MTG may also contribute to the adaptive processing of self-referential and emotionally salient information during therapy through interactions with large-scale networks such as the default mode and salience networks [54]. These mechanisms provide a plausible framework for understanding why individual differences in right MTG volume might be associated with CBT-related symptom improvement.

However, our exploratory analysis of cognitive versus affective-somatic BDI-II symptoms did not provide evidence for a domain-specific association with MTG volume. Thus, while the potential involvement of temporal cortical morphology in cognitive processes relevant to CBT remains an interesting possibility, the present findings do not establish a specific relationship between MTG structure and improvement in negative cognitions or other cognitive symptoms. Future studies incorporating more specific measures of negative beliefs, automatic thoughts, and rumination, as well as functional and connectivity-based approaches, could more directly test this proposed mechanism. Taken together, the findings suggest that structural preservation of the right MTG may serve as a key neural factor contributing to individual differences in CBT responsiveness, which was not attributable to differences in MRI reconstruction quality, highlighting the importance of temporal cortical regions beyond the limbic system traditionally emphasized in depression research.

Also, the MTG has been implicated in different psychiatric disorders before, such as obsessive-compulsive disorder [55], childhood maltreatment/PTSD [56], social anxiety disorder [57], and addiction disorders [53]. Another study highlighted its mediating role within the association between smartphone use and depressive symptoms in students [58]. The heterogeneity of treatment response also warrants consideration. In an exploratory comparison, patients whose symptoms worsened during treatment did not show consistent differences in demographic or clinical characteristics compared with those who improved, except for childhood maltreatment, a risk factor for treatment-resistant depression [59, 60], and, at trend-level, bilateral MTG volume. Larger samples will be needed to determine whether non-response or deterioration during CBT is associated with distinct neurobiological profiles.

The exploratory out-of-sample analysis provides a more cautious perspective on the potential utility of baseline cortical GMV. Although the combined model yielded modest out-of-sample performance, cortical volume measures provided limited additional predictive information beyond established clinical and demographic variables. This finding suggests that the association between baseline right MTG volume and subsequent symptom improvement should not be interpreted as evidence that MTG volume alone cannot allow accurate individual predictions. Rather, the finding may indicate that structural variation in the MTG captures individual differences relevant to symptom change.

We did not observe significant GMV differences between patients and HC at baseline after FDR correction. This is consistent with recent large-scale evidence indicating that structural cortical differences in depression are small in magnitude and inconsistent across samples [10, 11], particularly in outpatient populations with mild-to-moderate symptomatology, and suggests that reliable case-control structural effects typically require sample sizes considerably larger than those attainable in single-site clinical studies, including the present one. While other work has reported significant case-control differences [9], such findings often derive from larger or pooled samples better powered to detect effects of this magnitude; the present null result is therefore more plausibly attributable to limited power than to a genuine absence of structural alteration in this cohort. Nevertheless, within-group variability in cortical morphology appears to meaningfully relate to treatment outcomes, underscoring the value of examining individual differences in brain structure as predictors of clinical change, rather than relying solely on case-control contrasts to establish their relevance.

### Strengths and limitations

This study has several notable strengths, including matched samples without meaningful GMV differences, an innovative research question, and a sophisticated design in the still underexplored field of neuroimaging in major depressive disorder. Together, these features provide novel insights into neurobiological factors associated with CBT response. Furthermore, sensitivity analyses accounting for FreeSurfer-derived Euler numbers yielded comparable results, increasing confidence that the observed association was not explained by differences in cortical surface reconstruction quality. If replicated, right MTG volume could serve as a promising biomarker to inform treatment selection by helping identify patients most likely to benefit from CBT. More broadly, the findings may contribute to a more comprehensive understanding of individual differences in treatment response when considered alongside established clinical characteristics.

Several limitations should also be acknowledged. The sample size, while consistent with prior neuroimaging studies of psychotherapy, was modest and may limit generalizability. Structural MRI captures only brain morphology; future studies could incorporate functional and connectivity analyses to better elucidate the dynamic mechanisms underlying CBT-related improvement. Although a subset of participants exhibited worsening of depressive symptoms during CBT, the present sample was not sufficiently powered to characterize neurobiological differences between treatment responders and patients who fail to improve or deteriorate. Future studies with larger samples should specifically investigate these clinically important subgroups. Additionally, the absence of a patient control group (e.g., receiving alternative treatment or no intervention) limits the ability to draw conclusions about the specificity of the observed effects to CBT, as improvements may reflect more general treatment-related or time-related changes within MDD patients. Due to the comparably small sample size, replication in larger, independent cohorts is highly recommended. Finally, the observational design precludes causal inference; longitudinal imaging during therapy would strengthen causal interpretation.

### Conclusion

In conclusion, this study provides preliminary evidence that pre-treatment GMV of the right MTG is associated with symptom improvement following CBT in unipolar depression. These findings highlight temporal cortical regions as potentially relevant for understanding individual variability in treatment response and underscore the value of integrating neurobiological measures with clinical measures. Future research in larger, independent cohorts, incorporating multimodal neuroimaging and longitudinal designs, is warranted to replicate and extend these findings and to explore their translational utility in personalized treatment planning.

## Data Availability

The data underlying this article will be shared on reasonable request to the corresponding author.

## Supplemental information

Supplemental Material

## Code availability

The code underlying this article will be shared on reasonable request to the corresponding author.
