# Supplementary material for: Temporal cortical gray matter volume relates to symptom improvement during cognitive behavioral therapy for depression
Source: Transl Psychiatry. 2026 Nov 3;16:608. doi: 10.1038/s41398-026-04545-x (PMC13731642; doi:10.1038/s41398-026-04545-x)
Supplement: Supplementary file 1 [file 41398_2026_4545_MOESM1_ESM.docx]

Supplemental Material

# Supplemental Methods

## Model assumption checks

## For each of the 68 regional models examining associations between cortical GMV and symptom reduction, model assumptions were evaluated using residual diagnostics. Normality of residuals was assessed using the Shapiro-Wilk test, homoscedasticity using the studentized Breusch-Pagan test implemented in *lmtest* (v0.9.40; Zeileis & Hothorn, 2002), and multicollinearity using variance inflation factors (VIF) computed with *car* (v3.1.5; Fox & Weisberg, 2019) for all model predictors. Case influence was evaluated using Cook's distance, with values approaching or exceeding 1.0 considered indicative of a disproportionately influential observation (Cook & Weisberg, 1982).

## MRI reconstruction quality

Cortical surface reconstruction quality was quantified using the FreeSurfer Euler number, an established index of reconstruction quality derived from cortical surface topology (Rosen et al., 2018), with more negative values indicating more topological defects. Euler numbers were extracted separately for the left and right hemispheres, and a total Euler number was calculated as their sum. Group differences in left, right, and total Euler numbers were examined using Welch’s *t*-tests. In addition, the total Euler number was included as an additional covariate in the primary multiple regression model as a sensitivity analysis to evaluate whether the association between baseline right middle temporal gyrus (MTG) gray matter volume (GMV) and symptom improvement was robust to variation in MRI reconstruction quality.

## Exploratory analysis of depressive symptom domains

To explore whether the association between baseline right MTG volume and subsequent symptom improvement was preferentially related to cognitive symptoms, Beck’s Depression Inventory-II (BDI-II; Hautzinger et al., 2006) items were grouped into cognitive and affective-somatic domains based on the previously established two-factor structure reported by Beck (1996). Total reduction scores were calculated separately for each domain.

A linear mixed-effects model was then used to test whether the association between baseline right MTG volume and symptom change differed between the two symptom domains using the packages *lme4* (v2.0.6; Bates et al., 2015) and *lmerTest* (v3.2.1; Kuznetsova et al., 2017). The model included symptom domain, standardized right MTG volume, and their interaction, as well as age, gender, medication load index, and total intracranial volume as covariates. A subject-specific random intercept accounted for the repeated observations across symptom domains.

**Exploratory analysis of patients with symptom improvement versus worsening**

To examine whether patients whose depressive symptoms worsened during treatment differed from those who showed symptom improvement, an exploratory subgroup analysis was conducted. Patients were classified according to the direction of change in BDI-II total score from baseline to follow-up, with an increase in scores indicating symptom worsening. Between-group differences in demographics were examined using Welch’s t-tests for continuous variables and χ² tests for categorical variables. ANCOVAs corresponding to the primary clinical and cortical GMV analyses were additionally conducted to examine between-group differences in baseline measures while adjusting for age, gender, and, for GMV variables, total intracranial volume. Multiple comparisons across cortical regions were controlled using the false discovery rate (FDR).

# Supplemental Results

## Model assumption checks

VIF for the covariate block common to all 68 regional models (age, medication load index, total intracranial volume, and gender) were all <1.4, indicating no problematic multicollinearity among the core covariates (Kutner et al., 2005; O’Brien, 2007). Across all 68 regional models, residuals deviated significantly from normality in 4 models (5.9%), consistent with the false-positive rate expected under the null at α=.05, and no model showed evidence of heteroscedasticity (all Breusch-Pagan *p*>.05). Multicollinearity was not a concern in any model (all VIF<2.1; Kutner et al., 2005; O’Brien, 2007). Maximum Cook's distance did not exceed 0.79 in any of the 68 models, well below the conventional threshold of 1.0, indicating that no single case disproportionately influenced the results in any region.

For the right MTG model, which survived FDR correction (β=.55, *p*_FDR_=.033), residuals did not significantly deviate from normality (Shapiro-Wilk *W*=0.98, *p*=.382), there was no evidence of heteroscedasticity (Breusch-Pagan *BP*=3.66, *df*=5, *p*=.599), and all predictor VIFs were <1.7, indicating no problematic multicollinearity (Kutner et al., 2005; O’Brien, 2007). Maximum Cook's distance for this model was 0.43, with no case approaching the conventional influence threshold. Together, these diagnostics indicate that the association between right MTG volume and symptom improvement is not an artifact of model misspecification, outlier influence, or violated regression assumptions.

## MRI reconstruction quality

No significant differences in cortical reconstruction quality were observed between patients and healthy controls for the left hemisphere Euler number, right hemisphere Euler number, or total Euler number (Welch’s *t*-tests: p≥.368; Mann-Whitney *U* tests: *p*≥.178; see **Supplemental Table 2**).

Inclusion of total Euler number as an additional covariate did not materially alter the results. The association between baseline right MTG volume and subsequent symptom improvement remained significant (total reduction: β=.54, *p*_FDR_=.046; percentage reduction: β=.48, *p*_FDR_=.099). As in the main analysis, left MTG volume showed only a trend-level association with total and percentage symptom reduction (*p*_FDR_≥.271). No other regions showed significant associations (*p*_FDR_≥.099). These findings indicate that the observed association between right MTG volume and subsequent symptom improvement was robust to adjustment for differences in MRI reconstruction quality.

## Exploratory symptom-domain analysis

Baseline right MTG volume was associated with improvement in both BDI-II symptom domains, with numerically larger effect estimates for the cognitive domain (β=.56, *p*_FDR_=.034) than for the affective-somatic domain (β=.47, *p*_FDR_=.221). However, the interaction between right MTG volume and symptom domain was not significant (β=-.03, *p*=.736), indicating no evidence that the strength of the association differed between cognitive and affective-somatic symptoms.

**Patients with symptom worsening**

A total of *n*=12 patients showed an increase in BDI-II total scores from baseline to follow-up. Compared with patients who showed symptom improvement, these patients did not differ significantly in age, gender, or baseline BDI-II scores. Differences were observed for childhood trauma and follow-up BDI-II scores (see **Supplemental Table 3**). Exploratory comparisons of baseline cortical GMV also identified nominal group differences in the bilateral MTG and several other cortical regions; however, none remained significant after FDR correction (*p*_FDR_≥.073; **Supplemental Table 4**). Given the small number of patients showing symptom worsening, these findings should be considered exploratory.

## Additional group × time interaction analysis of depression symptom severity

The mixed-design ANOVA revealed a significant main effect of group on depressive symptom severity (F(1,92)=155.69, p<.001, ηp²=.629), indicating that groups differed substantially in overall BDI-II scores. A significant main effect of time was also observed (F(1,92)=25.12, p<.001, η_p_²=.214), reflecting a general decrease in depressive symptoms from baseline to follow-up across an average interval of *M*=27.70 weeks (*SD*=2.08). Importantly, the group × time interaction was significant (*F*(1,92)=17.10, *p*<.001, ƞ_p_²=.157), indicating that symptom changes over time differed between groups. Post-hoc pairwise comparisons based on estimated marginal means showed that MDD patients exhibited a significant reduction in depressive symptoms over the course of CBT (t(92)=6.84, p<.001), whereas no significant change was observed in the control group (t(92)=0.59, p=.56).

# Supplemental Tables

## Supplemental Table 1

*Nominal group differences in cortical gray matter volume measures and bilateral middle temporal gyrus volumes*

| **Region of interest** | **Hemisphere of cluster** | **MDD (*n* = 52)** | **HC (*n* = 42)** |  |  |
| --- | --- | --- | --- | --- | --- |
|  |  | ***M* (*SD*)** | ***M* (*SD*)** | **ANCOVA** | ***p*** |
| Caudal middle frontal gyrus^1^ | L | 6449.54 (860.84) | 6930.07 (1043.66) | *F*(1,89)=7.53 | .007 |
| Entorhinal cortex^1^ | R | 1821.48 (319.68) | 1956.64 (370.11) | *F*(1,89)=4.37 | .039 |
| Inferior parietal gyrus^1^ | R | 15566.98 (2149.23) | 16588.86 (2603.73) | *F*(1,89)=6.83 | .011 |
| Rostral anterior cingulate gyrus^1^ | R | 1963.83 (407.95) | 2153.48 (349.40) | *F*(1,89)=7.55 | .007 |
| Middle temporal gyrus^2^ | L | 11971.02 (1513.35) | 12374.40 (1619.08) | *F*(1,89)=2.83 | .096 |
| Middle temporal gyrus^2^ | R | 12973.23 (1565.90) | 13292.07 (1757.79) | *F*(1,89)=1.53 | .220 |

*Note*. Groups included depressive patients (MDD) and healthy controls (HC); clinical diagnoses were assessed using the German Structured Clinical Interview for DSM-5 disorders (Beesdo-Baum et al., 2019). Cortical GMV measures in mm^3^ were obtained using FreeSurfer (version 5.3; *recon-all* pipeline) with standard preprocessing and Desikan–Killiany atlas parcellation (Desikan et al., 2006) and compared between groups using analysis of covariance (ANCOVA) models adjusted for age, gender, and total intracranial volume.

¹ Nominal group differences (uncorrected *p*≤.05; not significant after FDR correction)

² Middle temporal gyrus volumes (no significant group differences)

## Supplemental Table 2

*Group comparisons of Euler numbers*

| **Region of interest** | **Hemisphere** | **MDD (*n* = 52)** | **HC (*n* = 42)** |  |  |
| --- | --- | --- | --- | --- | --- |
|  |  | ***M* (*SD*)** | ***M* (*SD*)** | ***t*-test** | ***p*** |
| Euler | L | -23.15 (13.84) | -25.48 (11.04) | *t*(91.99)=0.90 | .368 |
| Euler | R | -22.08 (9.96) | -21.24 (10.71) | *t*(84.94)=0.39 | .698 |
| Total Euler | - | -45.23 (20.85) | -46.71 (19.62) | *t*(89.82)=0.35 | .724 |

*Note*. Groups included depressive patients (MDD) and healthy controls (HC); clinical diagnoses were assessed using the German Structured Clinical Interview for DSM-5 disorders (Beesdo-Baum et al., 2019). Welch *t*-tests were used for group comparisons. Freesurfer’s automated recon-all pipeline was used. Euler numbers were extracted from orig.nofix.

## Supplemental Table 3

*Comparison of improved (n=40) versus worsened (n=12) patients*

|  |  |  | **Improved**  **(*n* = 40)** | **Worsened**  **(*n* = 12)** |  |  |  |
| --- | --- | --- | --- | --- | --- | --- | --- |
|  |  |  | ***M* (*SD*)** | ***M* (*SD*)** | **Group comparison** | ***p*** |  |
| **Sociodemographic characteristics** | | |  |  |  |  |  |
|  | Gender (f/m) | | 30/10 | 6/6 | χ^2^(1)=2.71 | .100 |  |
|  | Age | | 27.38 (7.26) | 31.42 (10.89) | *t*(14.06)=1.21 | .247 |  |
|  | Years of education | | 13.13 (2.11) | 12.75 (1.91) | *t*(19.79)=0.58 | .568 |  |
| **Longitudinal interval** | | |  |  |  |  |  |
|  | Time interval in weeks | | 27.86 (2.30) | 27.29 (1.04) | *t*(41.43)=1.21 | .233 |  |
|  | Number of CBT sessions | | 18.97 (9.06) | 18.83 (16.02) | *t*(13.24)=0.03 | .977 |  |
| **Clinical characteristics** | | |  |  |  |  |  |
|  | *Clinical questionnaire data* | |  |  |  |  |  |
|  |  | Depressive symptom severity at baseline | 24.05 (8.06) | 19.25 (10.18) | *F*(1,48)=2.79 | .101 |  |
|  |  | Depressive symptom severity at follow-up | 11.48 (8.40) | 25.67 (12.78) | *F*(1,48)=20.44 | <.001 |  |
|  |  | Anxiety | 55.43 (7.57) | 55.92 (6.52) | *F*(1,48)=0.04 | .842 |  |
|  |  | Perceived stress | 36.18 (4.28) | 34.42 (4.94) | *F*(1,48)=1.43 | .238 |  |
|  |  | Childhood trauma experiences | 38.68 (8.07) | 47.33 (15.72) | *F*(1,48)=6.61 | .013 |  |
|  | *Medication load index* | |  |  |  |  |  |
|  |  | 0 = absent | 33 (82.50%) | 9 (75.00%) | - | - |  |
|  |  | 1 = low | 7 (17.50%) | 2 (16.67%) | - | - |  |
|  |  | 2 = high | - | 1 (8.33%) | - | - |  |
|  | *DSM-V diagnosis* | |  |  |  |  |  |
|  |  | MDD, recurrent | 27 (67.50%) | 7 (58.33%) | - | - |  |
|  |  | MDD, single episode | 5 (12.50%) | 3 (25.00%) | - | - |  |
|  |  | Dysthymia | 7 (17.50%) | 2 (16.67%) | - | - |  |
|  |  | Other depressive disorder | 1 (2.50%) | - | - | - |  |
|  | *Degree of remission* | |  |  |  |  |  |
|  |  | In partial remission | 1 (2.50%) | 1 (8.33%) | - | - |  |
|  |  | Acute | 39 (97.50%) | 11 (91.67%) | - | - |  |
|  |  | Comorbidity (yes/no) | 22/18 | 9/3 | χ^2^(1)=1.53 | .216 |  |

*Note*. Groups comprised patients with symptom improvement and symptom worsening from baseline to follow-up. Clinical diagnoses were assessed using the German Structured Clinical Interview for DSM-5 disorders (Beesdo-Baum et al., 2019). Group differences in age, years of education, assessment interval, and number of cognitive behavioral therapy (CBT) sessions were assessed using Welch’s *t*-tests. All other continuous variables were compared between groups using analysis of covariance models adjusted for age and gender. Gender and comorbidity status were compared using a χ2-test. Depressive symptom severity was measured with the German Beck Depression Inventory-II (Hautzinger et al., 2006) at baseline and at follow-up after CBT, while additionally, only at baseline, trait anxiety was assessed using the German State-Trait Anxiety Inventory (Laux et al., 1981), perceived stress using the German Perceived Stress Scale (Schneider et al., 2020), and childhood trauma experiences using the German Childhood Trauma Questionnaire (Klinitzke et al., 2012).

## Supplemental Table 4

*Nominal group differences between improved (n=40) versus worsened (n=12) patients within MDD in cortical gray matter volume measures and Euler numbers*

| **Region of interest** | | **Hemisphere of cluster** | **Improved**  **(*n* = 40)** | **Worsened**  **(*n* = 12)** |  |  |
| --- | --- | --- | --- | --- | --- | --- |
|  |  |  | ***M* (*SD*)** | ***M* (*SD*)** | **Group comparison** | ***p*** |
| **Regions of interest** | |  |  |  |  |  |
|  | Bank of the superior temporal sulcus^1^ | L | 2718.95 (669.80) | 2301.33 (306.73) | *F*(1,47)=5.29 | .026 |
|  | Inferior temporal gyrus^1^ | L | 11870.27 (1417.19) | 10865.42 (1571.14) | *F*(1,47)=5.58 | .022 |
|  | Middle temporal gyrus^1^ | L | 12216.38 (1456.59) | 11153.17 (1465.44) | *F*(1,47)=10.38 | .002 |
|  | Middle temporal gyrus^1^ | R | 13285.95 (1447.62) | 9926.75 (1681.92) | *F*(1,47)=12.14 | .001 |
|  | Supramarginal gyrus^1^ | R | 11105.40 (1681.10) | 9926.75 (1681.92) | *F*(1,47)=5.10 | .029 |
|  | Transverse temporal cortex^1^ | R | 1053.90 (159.30) | 928.92 (188.29) | *F*(1,47)=6.23 | .016 |
| **Euler number** | |  |  |  |  |  |
|  | Euler | L | -23.45 (13.64) | -22.17 (15.05) | *t*(16.80)=0.26 | .795 |
|  | Euler | R | -21.30 (9.24) | -24.67 (12.13) | *t*(15.04)=0.89 | .389 |
|  | Total Euler | - | -44.75 (19.68) | -46.83 (25.28) | *t*(15.22)=0.26 | .796 |

*Note*. Groups comprised patients with symptom improvement and symptom worsening from baseline to follow-up. Clinical diagnoses were assessed using the German Structured Clinical Interview for DSM-5 disorders (Beesdo-Baum et al., 2019). Cortical GMV measures in mm^3^ were obtained using FreeSurfer (version 5.3; *recon-all* pipeline) with standard preprocessing and Desikan–Killiany atlas parcellation (Desikan et al., 2006), and were compared between groups using analysis of covariance (ANCOVA) models adjusted for age, gender, and total intracranial volume. Welch *t*-tests were used for group comparisons of Euler numbers resulting from FreeSurfer’s automated recon-all pipeline and extracted from orig.nofix.

¹ Nominal group differences (uncorrected *p*≤.05; not significant after FDR correction)

# References

Bates, D., Mächler, M., Bolker, B., & Walker, S. (2015). Fitting Linear Mixed-Effects Models Using lme4. *Journal of Statistical Software*, *67*, 1–48. https://doi.org/10.18637/jss.v067.i01

Beck, A. T., Steer, R. A., & Brown, G. K. (1996). *Manual for the Beck Depression Inventory-II*. Psychological Corporation.

Beesdo-Baum, K., Zaudig, M., & Wittchen, H.-U. (2019). *SCID-5-CV - Strukturiertes Klinisches Interview für DSM-5-Störungen* (1st ed.). Hogrefe.

Cook, R. D., & Weisberg, S. (1982). Criticism and Influence Analysis in Regression. *Sociological Methodology*, *13*, 313–361.

Desikan, R. S., Ségonne, F., Fischl, B., Quinn, B. T., Dickerson, B. C., Blacker, D., Buckner, R. L., Dale, A. M., Maguire, R. P., Hyman, B. T., Albert, M. S., & Killiany, R. J. (2006). An automated labeling system for subdividing the human cerebral cortex on MRI scans into gyral based regions of interest. *NeuroImage*, *31*(3), 968–980. https://doi.org/10.1016/j.neuroimage.2006.01.021

Fox, J., & Weisberg, S. (2019). *An R Companion to Applied Regression* (3rd ed.). Sage. https://www.john-fox.ca/Companion/

Hautzinger, M., Keller, F., Kühner, C., & Beck, A. T. (2006). *BDI-II. Beck-Depressions-Inventar Revision – Manual*. Harcourt Test Services.

Klinitzke, G., Romppel, M., Häuser, W., Brähler, E., & Glaesmer, H. (2012). The German Version of the Childhood Trauma Questionnaire (CTQ): Psychometric characteristics in a representative sample of the general population. *Psychotherapie, Psychosomatik, medizinische Psychologie*, *62*(2), 47–51. https://doi.org/10.1055/s-0031-1295495

Kutner, M. H., Nachtsheim, C. J., Neter, J., & Li, W. (2005). *Applied Linear Statistical Models* (5th ed.). McGraw-Hill/Irwin.

Kuznetsova, A., Brockhoff, P. B., & Christensen, R. H. B. (2017). lmerTest Package: Tests in Linear Mixed Effects Models. *Journal of Statistical Software*, *82*, 1–26. https://doi.org/10.18637/jss.v082.i13

Laux, L., Glanzmann, P., & Spielberger, C. D. (1981). *Das State-Trait-Angstinventar (STAI)* (1. Aufl.). Beltz.

O’Brien, R. M. (2007). A Caution Regarding Rules of Thumb for Variance Inflation Factors. *Quality & Quantity*, *41*(5), 673–690. https://doi.org/10.1007/s11135-006-9018-6

Rosen, A. F. G., Roalf, D. R., Ruparel, K., Blake, J., Seelaus, K., Villa, L. P., Ciric, R., Cook, P. A., Davatzikos, C., Elliott, M. A., Garcia de La Garza, A., Gennatas, E. D., Quarmley, M., Schmitt, J. E., Shinohara, R. T., Tisdall, M. D., Craddock, R. C., Gur, R. E., Gur, R. C., & Satterthwaite, T. D. (2018). Quantitative assessment of structural image quality. *NeuroImage*, *169*, 407–418. https://doi.org/10.1016/j.neuroimage.2017.12.059

Schneider, E. E., Schönfelder, S., Domke-Wolf, M., & Wessa, M. (2020). Measuring stress in clinical and nonclinical subjects using a German adaptation of the Perceived Stress Scale. *International Journal of Clinical and Health Psychology: IJCHP*, *20*(2), 173–181. https://doi.org/10.1016/j.ijchp.2020.03.004

Zeileis, A., & Hothorn, T. (2002). Diagnostic Checking in Regression Relationships. *R News*, *2*(3), 7–10.
